# Supplementary material for: Examining publication bias—a simulation-based evaluation of statistical tests on publication bias
Source: PeerJ. 2017 Nov 30;5:e4115. doi: 10.7717/peerj.4115 (PMC5712469; doi:10.7717/peerj.4115)
Supplement: Supplemental Information 1 [file peerj-05-4115-s001.zip › Codebook.pdf]

Examining publication bias – A simulation-based evaluation of statistical tests on publication bias

Andreas Schneck

Codebook of simulation dataset

group	variable name	description
process	reg_count	regressions executed per MA
	r_time	run-time MA in seconds
	run	run of simulation (1-4)
parameter	k	number of trials in MA
	pb	intent to commit PB
	trim	intend to commit trimming (p-hacking) instead of file-drawer
	het	degree of effect heterogeneity
	mu_n	sim. number of observation in trials
	es	effect size of trials
descriptive results	mean_N	actual number of observation in trials
	I_sq	degree of effect heterogeneity in I^2
	pb_int	actual share of intended PB
	pb_com	actual share of committed PB
	pb_suc	auctual share of successful PB
Test results	pu_p	p-uniform: p-value
	pu_N	p-uniform: number of included trials
	pet_t	FAT/PET: t-value of PET
	fat_t	FAT/PET: p-value of FAT
	ct3_p	caliper test 3%: p-value
	ct3_N	caliper test 3%: number of included trials
	ct3_P	caliper test 3%: proportion over-caliper
	ct5_p	caliper test 5%: p-value
	ct5_N	caliper test 5%: number of included trials
	ct5_P	caliper test 5%: proportion over-caliper
	ct10_p	caliper test 10%: p-value
	ct10_N	caliper test 10%: number of included trials
	ct10_P	caliper test 10%: proportion over-caliper
	ct15_p	caliper test 15%: p-value
	ct15_N	caliper test 15%: number of included trials
	ct15_P	caliper test 15%: proportion over-caliper
	tes_p	TES: p-value
	tes_pE	TES: number of expected significant results
	tes_pO	TES: number of observed significant results
