# Supplementary material for: Examining publication bias—a simulation-based evaluation of statistical tests on publication bias
Source: PeerJ. 2017 Nov 30;5:e4115. doi: 10.7717/peerj.4115 (PMC5712469; doi:10.7717/peerj.4115)
Supplement: Appendix — Contains a detailed discussion of the publication bias tests evaluated as well as by condition results of the simulation study. [file peerj-05-4115-s002.pdf]

Examining publication bias – A simulation-based evaluation of statistical tests on publication bias

Andreas Schneck

Online Appendix

The online appendix provides further insights into the methodology of the tests evaluated in the Monte Carlo simulation study. Furthermore, the false positive rates as well as the statistical power of each simulated condition are presented.

Statistical tests on publication bias in detail

In the following section four publication bias, the regression-based FAT (Egger et al. 1997; Stanley & Doucouliagos 2014), PU (van Aert et al. 2016; van Assen et al. 2015), an extended version of p-curve (Simonsohn et al. 2014a; Simonsohn et al. 2014b; Simonsohn et al. 2015), the TES (Ioannidis & Trikalinos 2007) and the CT (Gerber & Malhotra 2008a; Gerber & Malhotra 2008b) tests are discussed in detail.

All of these tests are applied mostly in a discipline-specific context: The FAT is routinely used in classical meta-analyses across all disciplines (cf. the Cochrae Handbook Higgins & Green 2008: 314), PU (for applications see Blázquez et al. 2017; Head et al. 2015; Simmons & Simonsohn 2017), as well as the TES (for applications see Francis 2012a; Francis 2012b; Francis 2012c; Francis 2012d; Francis 2012e; Francis 2013) are more widely used in psychology. The CT is in contrast mostly implemented in the general social sciences (for further applications in Sociology and Political Science see Auspurg & Hinz 2011; Auspurg et al. 2014; Berning & Weiß 2015; Gerber & Malhotra 2008a; Gerber & Malhotra 2008b; in Psychology see Hartgerink et al. 2016; Kühberger et al. 2014). The discipline-specific use of the tests is therefore to a certain degree path dependent on the practices involved in testing publication bias in the specific fields.

Funnel asymmetry test (FAT)

The first class of tests makes it possible to address publication bias by the association of the effect sizes and their variance. Because the variance *(se^2)* of an effect size in a primary study *(es)* is strongly related to the sample size, small studies with a low number of observations (*N*) show an increased variation of effects around the unobserved true effect. The larger the *N*, the smaller the variation and thus the more precise is the effect size of the study. Under publication bias small non-significant studies are mostly omitted, whereas small but precise effects with a large *N* still remain in the analysis. When this pattern for a small positive effect is represented through a scatterplot graph a typical inverted funnel-shaped pattern can be observed (called "funnel plot" Light & Pillemer 1984: 63-69). In the exemplary Figure A1 on the right, studies in the lower left side are missing because of publication bias with a preference for significant positive effects. On the left side, in contrast, a symmetric funnel with no publication bias is shown.

Figure A1 Funnel asymmetry test (FAT)

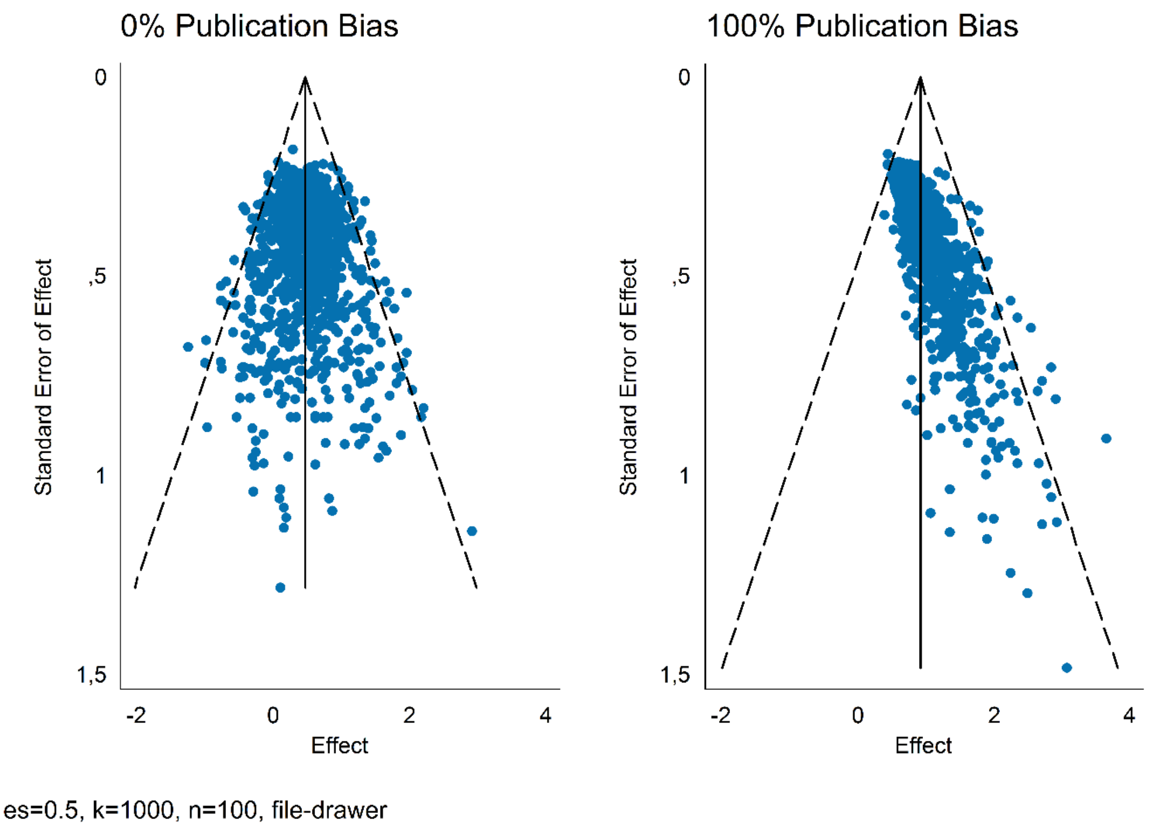

Exemplary funnel plot showing a symmetric funnel in the unbiased left graph and an asymmetric funnel in the right graph with an asymmetry towards positive effects.

Relying only on subjective graphical information, as provided by funnel plots, might be misleading (Tang & Liu 2000). Begg & Mazumdar (1994: 1089) examine the rank correlation of the standardised effect ($t = es/se$) and its variance (se^2). A similar approach by Egger et al. (1997)[1] regresses *t* on the inverse standard error ($1/se$). *t* is chosen as the dependent variable in order to account for the unequal variance across the effects (heteroscedasticity) by weighting each observation by the inverse of its variance. Compared to the regression of *se* on *es* this changes the interpretation.

[1] This estimator is equivalent to the bivariate FAT-PET recommended by Stanley & Doucouliagos (2014). The FAT-PET furthermore makes it possible to also include “potential effect modifiers” (Deeks et al. 2008: 284) in a meta-regression model. This is especially necessary if the literature being studied has, besides its theoretical meaningful overall effect, systematic differences (e.g. different implementations of an experimental stimulus, different experimental populations, etc.).

$$t_i = \beta_0 + \beta_1 \frac{1}{se_i} + \varepsilon_i$$

The constant β_0 is the test on publication bias (FAT stating publication bias if $\beta_0 \neq 0$), whereas β_1 makes it possible to identify a true empirical effect controlling for publication bias (Egger et al. 1997: 632). In the left graph of Figure A2 a primary study (depicted as one dot), with almost no precision, would not able to find an effect (H_0: $\beta_0 = 0$ could not be rejected). In contrast, in the right graph under publication bias a study with no precision would also find a substantial effect.

Figure A2 Funnel asymmetry test (FAT)

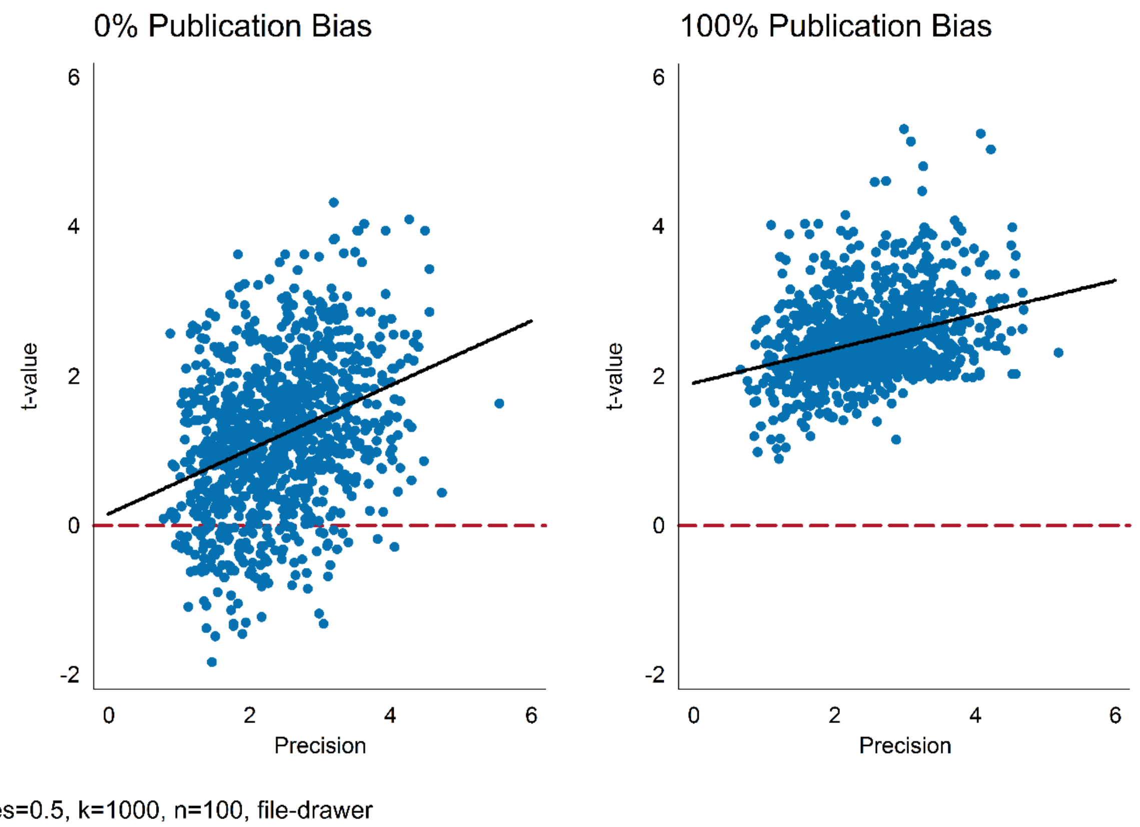

Exemplary graphical example of the FAT indicating no publication bias in the left (intercept through the origin) and publication bias in the right graph (positive intercept).

Despite its strengths, the central weaknesses of the FAT lies in its low statistical power in a setting with only a small number of primary studies (Macaskill et al. 2001 simmulated the performace only based on 20 primary studies).[2]

p-uniform (PU)

The tests discussed so far focus on the empirical effect sizes, whereas the p-curve method, proposed by Simonsohn et al. (2014b), and the similar PU, a method proposed by van Assen et al. (2015), focus entirely on the distribution of significant *p*-values. All non-significant values are therefore dropped from the analysis. The sample is, furthermore, restricted to the direction of suspected publication bias: that means only positive or negative effects are examined (Simonsohn et al. 2014a: 677). In the first step, the *p*-value of the estimate in the primary study is rescaled in respect to the significance threshold. For the present study the 5%-significance threshold (p = 0.05) rescales the *pp*-values to the range [0,1]. This *p*-value of *p*-values (*pp*-value) reflects the probability under the null hypothesis of a non-existing effect that a *p*-value would be as small as, or even smaller than, the observed one.[3]

$$pp_i = \frac{p_i}{0.05} = \frac{1 - \Phi\left(\frac{es_i}{se_i}\right)}{0.05} \quad if\ p_i < 0.05$$

In a second step the skewness of the *pp*-distribution is tested (Simonsohn et al. 2015: 1149). Right skewness shows an overrepresentation of findings with a substantial statistical significance and indicates a genuine empirical effect. Left skewness, in contrast, shows an overrepresentation of just significant estimates that barely pass the significance threshold (in this case 5%) and indicates publication bias under the null hypothesis (Simonsohn et al. 2014b: 536).

[2] In addition to the performance of the FAT, multiple simulation studies (Alinaghi & Reed 2016; Paldam 2015; Reed 2015) also examine the unbiasedness of the effect estimate (PET - the estimated underlying effect size corrected on publication bias) which is not of interest in the study at hand. The PET is especially threatened by an increased false positive rate under effect heterogeneity (Deeks et al. 2005; Stanley 2017), the properties of the FAT in these conditions have not yet been examined.

[3] Φ represents the standard normal distribution.

Whereas p-curve by Simonsohn et al. (2014b) only allows to identify publication bias under a true underlying null effect, PU (van Assen et al. 2015) allows to also identify publication bias under an empirically observed effect. This seems essential in order to distinguish between an underlying true effect and publication as criticised by Bruns & Ioannidis (2016) for p-curve. For PU as a first step the underlying effect has to be estimated empirically by a fixed-effect meta-analysis (FE-MA)[4] with all primary studies. In a second step, and equivalent to p-curve, only k estimates with $p < 0.05$ and the direction of the suspected publication bias remain in the analysis (van Aert et al. 2016: 727). By adjusting on the existing underlying effect, the fixed-effect estimate μ, it is possible to test the skewness of the distribution conditional on the underlying empirical effect (van Assen et al. 2015). In the case of a underlying null-effect, p-curve is therefore a special case of PU. In the numerator, the effect size estimate is conditioned on the underlying effect (μ), similar to a one-sample z-test. The denominator of the pp-value is not fixed to 0.05 as in p-curve, but is also conditioned on the underlying effect (μ), which is subtracted from the effect threshold (et) an effect has to reach to become statistically significant given its standard error (se).

$$pp_i^{\mu} = \frac{1 - \Phi\left(\frac{es_i - \mu}{se_i}\right)}{1 - \Phi\left(\frac{et_i - \mu}{se_i}\right)} \quad if\ p_i < 0.05$$

The test statistic is gamma-distributed with k degrees of freedom.[5] Because the skewness is now conditional on the underlying empirical effect left skewness observed by PU identifies publication bias across all underlying empirical effects, as depicted in Figure A3.

[4] Mean effect size across all included studies weighted by the inverse study variance.

[5] $p = \Gamma\left(k, -\sum_{i=1}^{k} \log(pp_i^{\mu})\right)$

Because PU rests on the average effect size estimated by a fixed-effects meta-analysis it may sensible to effect heterogeneity. The degree of heterogeneity which invalidates the publication bias test is, however, unclear for PU.[6]

Figure A3 p-uniform (PU)

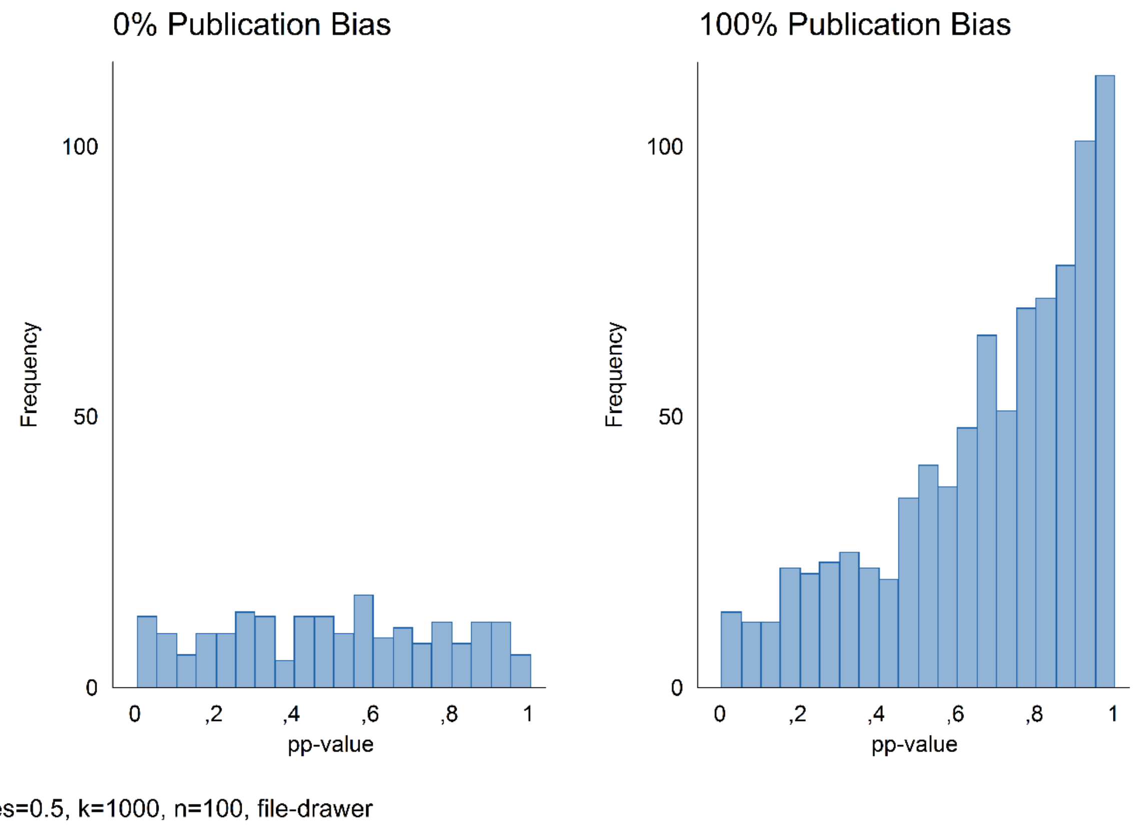

Exemplary graphical example of the PU indicating no publication bias in the left (uniform distribution of *pp*-values) and publication bias in the right graph (left skewed distribution of *pp*-values).

van Assen et al. (2015) evaluate the performance of PU, the TES (a publication bias test, discussed in the next section), as well as trim-and-fill, and conclude that PU has a greater statistical power than the other methods (van Assen et al. 2015: 303). Also, Renkewitz & Keiner (2016) evaluate the PU publication bias test and observe its slightly better performance compared to the FAT and the TES. However, in both studies the number of studies in the meta-

[6] Simonsohn et al. (2014a: 680) state that p-curve is able to estimate the average true effect of the observed significant studies correctly, whereas van Aert et al. (2016: 718) note the sensitivity towards heterogeneity of PU referring to the true underlying effect of all studies, which is mostly of concern in meta-analyses.

analyses (max. 160), as well as the number of observations (max. 80) in the primary studies, is relatively small.[7]

Test for excess significance (TES)

The TES builds on the observed power of every single study to uncover the true total effect. This true effect is estimated by a fixed-effect meta-analysis, as in PU. Observed power analyses make it possible to compute the post hoc power (pw_i) of a study. This allows to specify the expected number of significant effects E, given the average effect as well as the significance threshold (in this case $\alpha = 0.05$).[8]

$$E = \sum_{i=1}^{k} (pw_i)$$

E may even be a conservative estimate of the expected number of significant studies because it heavily relies on the fixed-effect estimate, which suffers from an eventual publication bias. In relation to O, the empirically observed number of significant studies ($p_i < 0.05$) the TES tests whether more significant results than expected are reported in the literature. To test whether the share of observed positive outcomes $\left(\frac{O}{K}\right)$ is larger than the share of expected positive outcomes $\left(\frac{E}{K}\right)$ a one-sided binomial test is used (Ioannidis & Trikalinos 2007: 246).

On exemplary datasets the TES performs considerably better under moderate effect heterogeneity in large meta-analyses, where the FAT in particular failed to uncover publication bias (Ioannidis & Trikalinos 2007: 248). Nevertheless, Johnson & Yuan (2007: 254) ask if the TES makes it possible to dissect between publication bias and study-heterogeneity accurately.

[7] Similar to the FAT-PET, evaluations of PU center mainly on the estimated overall effect. While van Assen et al. (2015) show a good coverage of the estimated overall effect, McShane et al. (2016) state, in contrast, that while "p-curve and p-uniform approaches have increased awareness about the consequences of publication bias in meta-analysis, they fail to improve upon, and indeed are inferior to, methods proposed decades ago" (McShane et al. 2016: 744).

[8] Although Hoenig & Heisey (2001) criticise the application of post-hoc power analyses in primary studies for the good reason that the observed power estimate may be biased, meta-analyses circumvent this critique because a distribution of power estimates allows to infer more accurately the power of a set of studies.

Therefore, the authors of the *Cochrane Handbook* (Higgins & Green 2008: 323) express the need for further evaluations.

Caliper test (CT)

In contrast to the aforementioned three tests, the CT, developed by Gerber and Malhotra (2008a; 2008b) ignores most of the information provided by the studies included and looks only at a narrow interval (caliper = *c*) around the significance threshold (*th*) in a distribution of absolute *z*-values. In case of a continuous distribution of *z*-values, studies in the interval below the significance threshold (in the so-called over-caliper; $x_z = 1$) should be as likely as just non-significant studies (in the so-called under-caliper; $x_z = 0$).

$$x_z = \begin{cases} 0 \; if \; th - c * th < z \leq th \\ 1 \; if \; th < z < th + c * th \end{cases}$$

Gerber and Malhotra (2008a; 2008b) use a 5%, 10%, 15% and 20% interval (*c*) proportional to the significance threshold (*th*). In particular, the widest 20% caliper may be too wide because the 10%-significance level that could be another target threshold for publication bias is fully overlapped. The higher the overrepresentation in the over-caliper, the higher the likelihood of publication bias. This is also shown in Figure A4: in the left graph with no publication bias no discontinuities are seen around the arbitrary 5% significance threshold (dashed line), whereas in the right graph a stepwise increase of just significant results indicates publication bias. As with the TES, a one-sided binomial test is used to test the equal distribution of *z*-values in the over- and under-caliper.[9]

[9] Masicampo & Lalande (2012) and Leggett et al. (2013) test the deviance of values around the significance threshold from a fitted exponential curve on *p*-values in a broader range from 0.1 – 0.10 to counter the huge loss of observations in the CT. This may be problematic, because a single distributive function may not be able to describe the pattern well enough across the suspected jump points (cf. Lakens 2015). In the case of substantial effect heterogeneity this problem would be aggravated even further.

Figure A4 Caliper test (CT with 5% caliper)

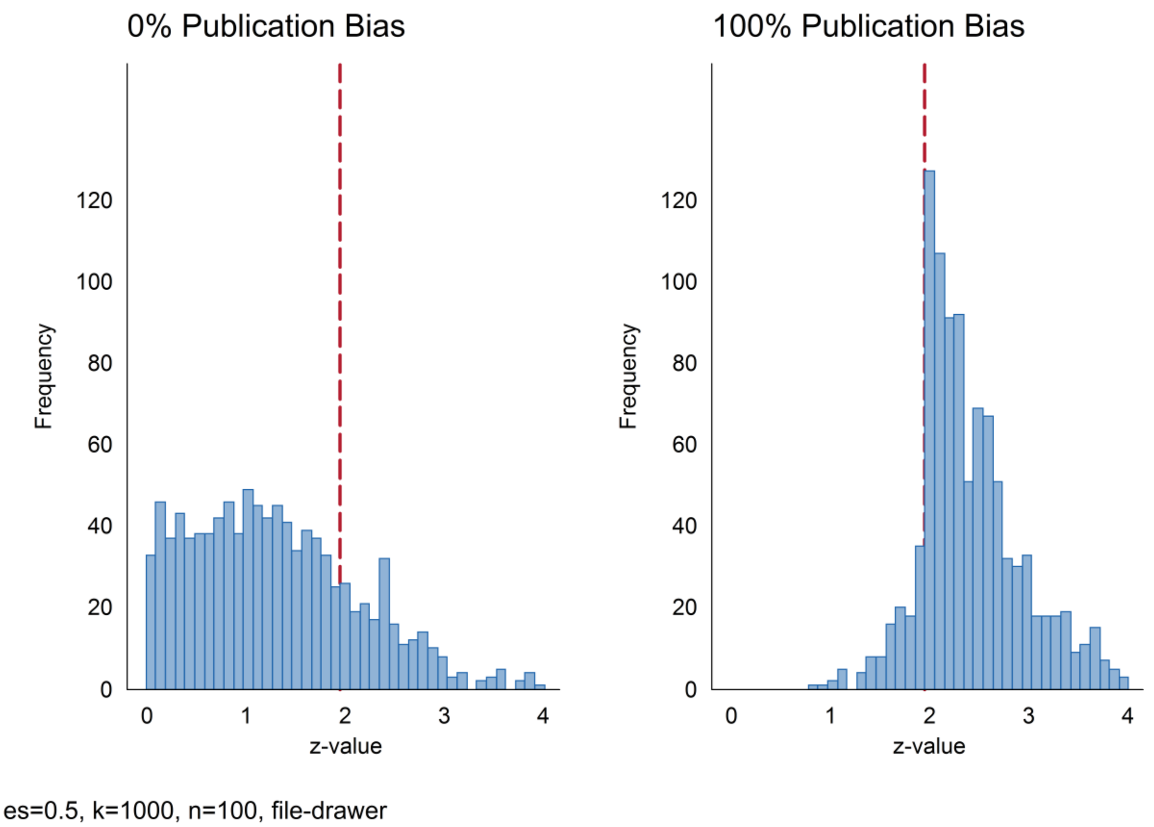

Exemplary graphical example of the CT indicating no publication bias in the left (no jump point around the significance threshold visualized by the red dashed line) and publication bias in the right graph (jump point at the 5% significance level).

Results in detail by simulation conditions

The following section presents the results of the false positive rates by each simulation condition. Besides the statistical power (Table A2-A5) of the evaluated publication bias tests also the actual committed as well as successful publication bias is reported along the results. As in the regression analysis in the article also the impact of publication bias on the meta-analytical *p*-value is reported.

False positive rates

Table A1 shows the false positive rates of the publication bias tests across all simulated conditions. Inflated false positive rates are highlighted in bold. Over all conditions the FAT, PU, the TES, as well as the narrower CTs (3%, 5%), had a consistent false positive rate. The FAT was closest to the expected 5% error rate. PU and the TES, as well as the 3% and 5% CTs,

in contrast, were in most cases very conservative because they fall far below 0.05. This over-conservatism may be problematic in respect to a decreased statistical power, a matter which is discussed later on. The wider 10% and 15% CTs suffered under inflated false positive rates because, due to the large caliper width, the assumption of a uniform distribution in both calipers was violated.[10] For the 10% CT the specified false positive rate doubles to more than 10%, whereas in case of the 15% CT it more than quadruples.

Table A1 False positive rates by each simulation condition

0% FD/PH	PU	FAT	TES	3% CT	5% CT	10% CT	15% CT
N100/K100							
0.0	0.045	0.043	0.024	0.001	0.001	0.003	0.002
0.5	0.039	0.045	0.004	0.004	0.011	0.013	0.012
1.0	0.014	0.056	0.005	0.005	0.017	0.033	0.040
1.5	0.001	0.047	0.010	0.000	0.005	0.026	0.041
Het	0.000	0.042	0.001	0.002	0.012	0.021	0.025
N100/K1000							
0.0	0.032	0.051	0.036	0.020	0.012	0.000	0.000
0.5	0.020	0.046	0.005	0.031	0.023	0.007	0.001
1.0	0.008	0.048	0.003	0.043	0.049	**0.067**	**0.092**
1.5	0.002	0.046	0.013	0.040	0.049	**0.101**	**0.204**
Het	0.000	0.047	0.000	0.032	0.032	0.028	0.030
N500/K100							
0.0	0.051	0.051	0.024	0.000	0.002	0.003	0.002
0.5	0.025	0.050	0.002	0.010	0.019	0.039	0.043
1.0	0.000	0.045	0.007	0.000	0.000	0.001	0.010
1.5	0.000	0.047	0.000	0.000	0.000	0.000	0.000
Het	0.000	0.037	0.000	0.000	0.002	0.011	0.018
N500/K1000							
0.0	0.043	0.052	0.037	0.019	0.009	0.001	0.000
0.5	0.024	0.042	0.004	0.039	0.045	**0.070**	**0.104**
1.0	0.000	0.054	0.033	0.018	0.043	**0.108**	**0.244**
1.5	0.000	0.048	0.003	0.000	0.000	0.002	0.007
Het	0.000	0.035	0.000	0.031	0.036	0.038	0.035

Bold numbers > 0.05 at $p < 0.05$

False positive rates of the seven evaluated tests by each condition. The 10% and 15% CT show an increased false positive rate (highlighted in bold).

[10] This means that an asymmetry between over- and under-caliper is not caused by publication bias rather than by an underlying effect distribution that is skewed in the caliper width.

Statistical power

Looking at conditions with 50% publication bias in the *file-drawer* condition (see Table A2), the FAT had a superior power compared to other tests in 14 of 20 conditions, as indicated by the underlined numbers. The FAT is, however, closely followed by the TES, which had a larger number of conditions with a satisfactory power (> 0.8) compared to the FAT (7 vs. 6). In the first condition with $N = 100$ as well as $K = 100$ the TES was superior in the case of an underlying small or moderate effect ($\beta = 0.5$; 1; 1.5). The large variability of the primary study effect, which was caused by the low-N and low-K in the meta-analyses, resulted in an overall minor statistical power. A sufficient power (highlighted in bold) was only reached in conditions with a low or moderate underlying true effect ($\beta = 0.5$, 1). This is caused by high prevalence of committed publication bias (PB com) that is also successful (PB suc – meaning $p < 0.05$). None of the CTs yielded a sufficient power. This picture changes if more studies were included in the meta-analysis. With $K = 1000$ most of the tests yielded a sufficient power. In particular, the FAT had a statistical power close to 100%, also under effect heterogeneity. The PU and the TES failed to uncover *file-drawer* behaviour under effect heterogeneity, but performed well under homogeneity. PU was only able to discover *file-drawer* behaviour under low underlying true effects. The CTs profited the most from an increased K, the wider caliper (10, 15%) had a larger statistical power than the narrower ones but also had inflated false positive rates (see Table A2) that might invalidate the conclusions (grey shaded area). The narrower caliper had a sufficient power only in studies with no or small underlying effects ($\beta = 0$; 0.5). $K = 100$ and $N = 500$ decreased the power of all tests drastically. In this condition the FAT had the largest, but still not satisfactory power. With $K = 1000$ a sufficient power is yielded in conditions with a low overall effect ($\beta = 0$; 0.5).

Table A2 Statistical power by each simulation condition for 50% file-drawer publication bias

50% FD	PU	FAT	TES	3% CT	5% CT	10% CT	15% CT	PB com	PB suc	*p* defl.
N100/K100										
0.0	0.179	0.662	0.148	0.007	0.013	0.015	0.005	0.488	0.215	0.154
0.5	0.691	**0.822**	**0.912**	0.108	0.220	0.416	0.563	0.379	0.843	0.070
1.0	0.348	**0.823**	**0.881**	0.052	0.149	0.415	0.594	0.184	0.977	0.068
1.5	0.034	0.457	0.537	0.007	0.032	0.164	0.285	0.073	0.997	0.260
Het	0.000	0.370	0.042	0.032	0.082	0.220	0.321	0.223	0.746	0.360
N100/K1000										
0.0	0.720	**1.000**	0.737	0.029	0.019	0.001	0.000	0.487	0.215	0.000
0.5	**1.000**	**1.000**	**1.000**	**0.894**	**0.981**	**1.000**	**1.000**	0.379	0.843	0.000
1.0	**1.000**	**1.000**	**1.000**	**0.859**	**0.976**	**0.999**	**1.000**	0.185	0.978	0.000
1.5	0.521	**0.999**	**1.000**	0.530	0.763	**0.981**	**0.999**	0.074	0.998	0.000
Het	0.000	**0.997**	0.125	0.639	**0.839**	**0.977**	**0.996**	0.224	0.746	0.002
N500/K100										
0.0	0.238	0.245	0.104	0.007	0.010	0.013	0.003	0.488	0.207	0.466
0.5	0.580	0.499	0.736	0.080	0.201	0.442	0.671	0.204	0.993	0.268
1.0	0.001	0.110	0.039	0.000	0.001	0.003	0.021	0.013	0.998	0.752
1.5	0.000	0.056	0.000	0.000	0.000	0.000	0.000	0.001	0.994	0.944
Het	0.000	0.058	0.000	0.005	0.029	0.095	0.166	0.116	0.748	0.836
N500/K1000										
0.0	**0.905**	**0.950**	0.544	0.043	0.028	0.001	0.000	0.487	0.207	0.019
0.5	**1.000**	**0.999**	**1.000**	**0.911**	**0.987**	**1.000**	**1.000**	0.205	0.992	0.000
1.0	0.004	0.396	**0.874**	0.068	0.165	0.529	**0.826**	0.013	0.998	0.328
1.5	0.001	0.064	0.019	0.000	0.000	0.005	0.019	0.001	1.000	0.887
Het	0.000	0.214	0.000	0.373	0.569	**0.855**	**0.950**	0.116	0.745	0.506
Best / Satisfactory	3 / 4	14 / 6	8 / 7	0 / 3	0 / 4	2 / 6	3 / 7			

Bold numbers: > 0.8 p < 0.05. Underlined: best estimator. Grey shaded: inflated false positive rate, cf. Table A1

PB com displays the share of studies committing publication bias. PB suc describes the share of studies successfully committing publication bias. *p* defl. shows the the deflation of the meta-analytical *p*-value by publication bias.

The statistical power of the tests increased if the intent to engage in *file-drawer* behaviour is set to 100% (see Table A3). Overall, more publication bias tests achieved a satisfactory statistical power to detect publication bias. Also, in these conditions, the FAT dominated in 13 of 20 conditions. As before, neither the TES nor the PU were able to detect publication bias under effect heterogeneity. The TES was, furthermore, not able to detect publication bias with an underlying null effect, despite publication bias was successfully applied by 21.3% of the cases. Similar to the 50% *file-drawer* condition, the CTs showed a drastically decreased power in conditions with $K = 100$.

Table A3 Statistical power by each simulation condition for 100% file-drawer publication bias

100% FD	PU	FAT	TES	3% CT	5% CT	10% CT	15% CT	PB com	PB suc	*p* defl.
N100/K100										
0.0	0.756	**1.000**	0.000	0.013	0.016	0.012	0.005	0.974	0.213	0.000
0.5	**1.000**	**1.000**	**1.000**	0.328	0.569	**0.891**	**0.981**	0.759	0.843	0.000
1.0	**0.958**	**1.000**	**1.000**	0.222	0.618	**0.962**	**0.999**	0.371	0.978	0.000
1.5	0.177	**0.975**	**1.000**	0.028	0.138	0.621	**0.898**	0.149	0.998	0.012
Het	0.000	**0.962**	**0.882**	0.124	0.278	0.595	0.790	0.447	0.746	0.015
N100/K1000										
0.0	**1.000**	**1.000**	0.000	0.047	0.021	0.002	0.000	0.975	0.215	0.000
0.5	**1.000**	**1.000**	**1.000**	**1.000**	**1.000**	**1.000**	**1.000**	0.759	0.843	0.000
1.0	**1.000**	**1.000**	**1.000**	**1.000**	**1.000**	**1.000**	**1.000**	0.369	0.978	0.000
1.5	**0.999**	**1.000**	**1.000**	**0.999**	**1.000**	**1.000**	**1.000**	0.149	0.998	0.000
Het	0.000	**1.000**	**1.000**	**0.981**	**0.999**	**1.000**	**1.000**	0.448	0.745	0.000
N500/K100										
0.0	**0.888**	**0.990**	0.000	0.011	0.012	0.015	0.003	0.975	0.208	0.005
0.5	**1.000**	**0.999**	**1.000**	0.351	0.755	**0.992**	**1.000**	0.410	0.992	0.001
1.0	0.001	0.221	0.235	0.000	0.000	0.006	0.047	0.026	0.998	0.527
1.5	0.001	0.059	0.000	0.000	0.000	0.000	0.000	0.002	0.997	0.931
Het	0.000	0.129	0.000	0.026	0.092	0.290	0.473	0.230	0.741	0.666
N500/K1000										
0.0	**1.000**	**1.000**	0.000	0.039	0.021	0.003	0.000	0.975	0.206	0.000
0.5	**1.000**	**1.000**	**1.000**	**1.000**	**1.000**	**1.000**	**1.000**	0.409	0.992	0.000
1.0	0.093	**0.898**	**1.000**	0.233	0.669	**0.995**	**1.000**	0.026	0.998	0.042
1.5	0.000	0.108	0.145	0.000	0.000	0.009	0.041	0.002	1.000	0.757
Het	0.000	0.628	0.000	**0.829**	**0.957**	**1.000**	**1.000**	0.231	0.743	0.153
Best / Satisfactory	7 / 10	13 / 15	12 / 11	3 / 6	4 / 6	6 / 10	9 / 11			

Bold numbers: > 0.8 p < 0.05. Underlined: best estimator. Grey shaded: inflated false positive rate, cf. Table A1

PB com displays the share of studies committing publication bias. PB suc describes the share of studies successfully committing publication bias. *p* defl. shows the the deflation of the meta-analytical *p*-value by publication bias.

The dominance of the FAT weakened when looking at the 50% *p-hacking* condition (see Table A4). Instead, the TES was besides the 15% CT superior under most conditions but had the advantage that its false positive rate was not inflated. The overall pattern was, however, quite similar: both PU and TES had almost no power to detect *p-hacking* under effect heterogeneity. Also, the statistical power was only satisfactory for PU when $K = 100$. With a large number of included studies, however, the power of the CT was close to, or even outperformed, the FAT, PU and the TES.

Table A4 Statistical power by each simulation condition for 50% p-hacking publication bias

50% PH	PU	FAT	TES	3% CT	5% CT	10% CT	15% CT	PB com	PB suc	*p* defl.
N100/K100										
0.0	0.764	0.006	0.598	0.077	0.139	0.196	0.166	0.489	0.305	1.331
0.5	**0.870**	0.371	0.490	0.152	0.288	0.465	0.527	0.379	0.535	0.357
1.0	0.321	0.395	0.422	0.079	0.168	0.396	0.528	0.184	0.598	0.354
1.5	0.018	0.129	0.166	0.015	0.058	0.154	0.259	0.074	0.602	0.693
Het	0.000	0.369	0.020	0.068	0.139	0.292	0.380	0.223	0.507	0.356
N100/K1000										
0.0	**1.000**	0.000	**1.000**	0.767	**0.874**	**0.929**	**0.846**	0.488	0.304	1.872
0.5	**1.000**	**0.992**	**1.000**	**0.973**	**0.995**	**1.000**	**1.000**	0.379	0.539	0.003
1.0	**0.997**	**0.997**	**1.000**	**0.879**	**0.968**	**0.999**	**1.000**	0.184	0.597	0.003
1.5	0.175	0.503	**0.962**	0.505	0.733	**0.958**	**0.994**	0.074	0.598	0.233
Het	0.000	**0.994**	0.007	0.797	**0.942**	**0.997**	**0.999**	0.224	0.507	0.004
N500/K100										
0.0	**0.958**	0.000	**1.000**	0.211	0.394	0.659	0.769	0.491	0.684	1.969
0.5	0.806	0.437	**0.843**	0.112	0.285	0.602	0.784	0.206	0.925	0.319
1.0	0.000	0.066	0.028	0.000	0.001	0.013	0.046	0.013	0.894	0.874
1.5	0.001	0.058	0.000	0.000	0.000	0.000	0.000	0.001	0.736	0.969
Het	0.000	0.408	0.000	0.015	0.067	0.233	0.383	0.115	0.843	0.310
N500/K1000										
0.0	**1.000**	0.000	**1.000**	**0.995**	**1.000**	**1.000**	**1.000**	0.488	0.685	2.011
0.5	**1.000**	**0.999**	**1.000**	**0.966**	**0.999**	**1.000**	**1.000**	0.204	0.923	0.001
1.0	0.004	0.159	0.775	0.116	0.271	0.676	**0.908**	0.013	0.886	0.645
1.5	0.000	0.046	0.012	0.002	0.002	0.007	0.026	0.001	0.749	0.972
Het	0.000	**0.997**	0.000	0.772	**0.935**	**0.998**	**1.000**	0.115	0.846	0.001
Best / Satisfactory	6 / 7	4 / 5	7 / 8	0 / 4	1 / 7	3 / 8	11 / 8			

Bold numbers: > 0.8 p < 0.05. Underlined: best estimator. Grey shaded: inflated false positive rate, cf. Table A1

PB com displays the share of studies committing publication bias. PB suc describes the share of studies successfully committing publication bias. *p* defl. shows the the deflation of the meta-analytical *p*-value by publication bias.

In the 100% *p-hacking* condition (see Table A5) the FAT caught up with the TES and yielded an increased power, especially in the case of K = 100. Despite the dominance of the 15% CT, the TES and the FAT closely followed. The CT had a similar strength to that demonstrated in the earlier conditions under effect heterogeneity and K = 1000. The underperformance of all tests in the condition with N = 500 and moderate underlying effects (β = 1; 1.5) is caused by the already existing significance of most results in this condition.

Table A5 Statistical power by each simulation condition for 100% p-hacking publication bias

100% PH	PU	FAT	TES	3% CT	5% CT	10% CT	15% CT	PB com	PB suc	*p* defl.
N100/K100										
0.0	**0.999**	0.808	0.212	0.203	0.331	0.477	0.497	0.975	0.305	0.079
0.5	**1.000**	**0.997**	**0.992**	0.481	0.727	**0.918**	**0.964**	0.759	0.536	0.002
1.0	**0.854**	**0.916**	**0.985**	0.286	0.518	**0.835**	**0.947**	0.368	0.596	0.032
1.5	0.089	0.293	0.679	0.051	0.165	0.443	0.648	0.149	0.606	0.419
Het	0.000	**0.903**	0.390	0.235	0.436	0.724	**0.847**	0.450	0.506	0.039
N100/K1000										
0.0	**1.000**	**1.000**	**0.999**	**0.984**	**0.999**	**1.000**	**1.000**	0.975	0.305	0.000
0.5	**1.000**	**1.000**	**1.000**	**1.000**	**1.000**	**1.000**	**1.000**	0.758	0.538	0.000
1.0	**1.000**	**1.000**	**1.000**	**1.000**	**1.000**	**1.000**	**1.000**	0.369	0.595	0.000
1.5	**0.887**	**0.976**	**1.000**	**0.957**	**0.997**	**1.000**	**1.000**	0.148	0.599	0.009
Het	0.000	**1.000**	**0.999**	**0.997**	**1.000**	**1.000**	**1.000**	0.447	0.507	0.000
N500/K100										
0.0	**1.000**	**0.979**	**1.000**	0.561	0.791	**0.977**	**0.994**	0.975	0.685	0.010
0.5	**0.999**	**0.997**	**1.000**	0.525	**0.847**	**0.995**	**1.000**	0.411	0.923	0.001
1.0	0.001	0.106	0.138	0.000	0.002	0.036	0.119	0.026	0.897	0.741
1.5	0.000	0.051	0.000	0.000	0.000	0.000	0.000	0.002	0.746	0.976
Het	0.000	**0.916**	0.003	0.099	0.328	0.758	**0.917**	0.231	0.847	0.032
N500/K1000										
0.0	**1.000**	**1.000**	**1.000**	**1.000**	**1.000**	**1.000**	**1.000**	0.975	0.685	0.000
0.5	**1.000**	**1.000**	**1.000**	**1.000**	**1.000**	**1.000**	**1.000**	0.409	0.923	0.000
1.0	0.028	0.405	**1.000**	0.438	0.804	**0.996**	**1.000**	0.026	0.885	0.310
1.5	0.000	0.061	0.028	0.000	0.002	0.022	0.072	0.002	0.739	0.953
Het	0.000	**1.000**	0.000	**0.998**	**1.000**	**1.000**	**1.000**	0.231	0.845	0.000
Best / Satisfactory	8 / 11	7 / 14	9 / 12	4 / 8	6 / 9	8 / 13	12 / 15			

Bold numbers: > 0.8 p < 0.05. Underlined: best estimator. Grey shaded: inflated false positive rate, cf. Table A1

PB com displays the share of studies committing publication bias. PB suc describes the share of studies successfully committing publication bias. *p* defl. shows the the deflation of the meta-analytical *p*-value by publication bias.

Overall, the FAT dominated under the *file-drawer* condition. The TES, in contrast, had a slightly higher statistical power than the FAT under the *p-hacking* condition without effect heterogeneity. However, the differences between both tests were quite small. The CTs performed well under the *file-drawer* as well as *p-hacking* condition with heterogeneous effect sizes and large numbers of studies included (K = 1000). Although the 10% and 15% caliper had the highest power to detect *p-hacking* these tests should not be applied due to their increased false positive rate.

Reference

Alinaghi N, and Reed WR. 2016. Meta-Analysis and Publication Bias: How Well Does the FAT-PET-PEESE Procedure Work? https://ideas.repec.org/p/cbt/econwp/16-26.html.

Auspurg K, and Hinz T. 2011. What Fuels Publication Bias? Theoretical and Empirical Analyses of Risk Factors Using the Caliper Test. *Journal of Economics and Statistics* 231:636-660.

Auspurg K, Hinz T, and Schneck A. 2014. Ausmaß und Risikofaktoren des Publication Bias in der deutschen Soziologie. *Koelner Zeitschrift fuer Soziologie und Sozialpsychologie* 66:549-573. 10.1007/s11577-014-0284-3

Begg CB, and Mazumdar M. 1994. Operating Characteristics of a Bank Correlation Test for Publication Bias. *Biometrics* 50:1088-1101. 10.2307/2533446

Berning CC, and Weiß B. 2015. Publication Bias in the German Social Sciences: an Application of the Caliper Test to Three Top-Tier German Social Science Journals. *Quality & Quantity* 50:901-917. 10.1007/s11135-015-0182-4

Blázquez D, Botella J, and Suero M. 2017. The Debate on the Ego-Depletion Effect: Evidence from Meta-Analysis with the p-Uniform Method. *Frontiers in Psychology* 8:197. 10.3389/fpsyg.2017.00197

Bruns SB, and Ioannidis JPA. 2016. p-Curve and p-Hacking in Observational Research. *PloS one* 11:e0149144. 10.1371/journal.pone.0149144

Deeks JJ, Higgins JPT, and Altman DG. 2008. Analysing data and undertaking meta-analyses. *Cochrane handbook for systematic reviews of interventions*: John Wiley & Sons, Ltd, 243-296.

Deeks JJ, Macaskill P, and Irwig L. 2005. The Performance of Tests of Publication Bias and Other Sample Size Effects in Systematic Reviews of Diagnostic Test Accuracy Was Assessed. *Journal of Clinical Epidemiology* 58:882-893. 10.1016/j.jclinepi.2005.01.016

Egger M, Smith GD, Schneider M, and Minder C. 1997. Bias in Meta-Analysis Detected by a Simple, Graphical Test. *British Medical Journal* 315:629-634.

Francis G. 2012a. Evidence That Publication Bias Contaminated Studies Relating Social Class and Unethical Behavior. *Proceedings of the National Academy of Sciences of the United States of America* 109:E1587-E1587. 10.1073/pnas.1203591109

Francis G. 2012b. The Psychology of Replication and Replication in Psychology. *Perspectives on Psychological Science* 7:585-594. 10.1177/1745691612459520

Francis G. 2012c. Publication Bias and the Failure of Replication in Experimental Psychology. *Psychonomic Bulletin & Review* 19:975-991. 10.3758/s13423-012-0322-y

Francis G. 2012d. The Same Old New Look: Publication Bias in a Study of Wishful Seeing. *i-Perception* 3:176-178. 10.1068/i0519ic

Francis G. 2012e. Too Good to Be True: Publication Bias in Two Prominent Studies from Experimental Psychology. *Psychonomic Bulletin & Review* 19:151-156. 10.3758/s13423-012-0227-9

Francis G. 2013. Publication Bias in 'Red, Rank, and Romance in Women Viewing Men,' by Elliot et al. (2010). *Journal of Experimental Psychology: General* 142:292-296. 10.1037/a0027923

Gerber AS, and Malhotra N. 2008a. Do Statistical Reporting Standards Affect What Is Published? Publication Bias in Two Leading Political Science Journals. *Quarterly Journal of Political Science* 3:313-326. 10.1561/100.00008024

Gerber AS, and Malhotra N. 2008b. Publication Bias in Empirical Sociological Research. *Sociological Methods & Research* 37:3-30. 10.1177/0049124108318973

Hartgerink CHJ, van Aert RCM, Nuijten MB, Wicherts JM, and van Assen MALM. 2016. Distributions of p-values smaller than .05 in psychology: what is going on? *Peerj* 4:e1935. 10.7717/peerj.1935

Head ML, Holman L, Lanfear R, Kahn AT, and Jennions MD. 2015. The Extent and Consequences of P-Hacking in Science. *PLoS Biol* 13:e1002106. 10.1371/journal.pbio.1002106

Higgins JPT, and Green S. 2008. *Cochrane Handbook for Systematic Reviews of Interventions*: Wiley Online Library.

Hoenig JM, and Heisey DM. 2001. The Abuse of Power. *The American Statistician* 55:19-24. 10.1198/000313001300339897

Ioannidis JPA, and Trikalinos TA. 2007. An Exploratory Test for an Excess of Significant Findings. *Clinical Trials* 4:245-253. 10.1177/1740774507079441

Johnson V, and Yuan Y. 2007. Comments on `An Exploratory Test for an Excess of Significant Findings' by JPA loannidis and TA Trikalinos. *Clinical Trials* 4:254-255. 10.1177/1740774507079437

Kühberger A, Fritz A, and Scherndl T. 2014. Publication Bias in Psychology: A Diagnosis Based on the Correlation between Effect Size and Sample Size. *PloS one* 9:e105825. 10.1371/journal.pone.0105825

Lakens D. 2015. What R-hacking Really Looks Like: A Comment on Masicampo and LaLande (2012). *The Quarterly Journal of Experimental Psychology* 68:829-832. 10.1080/17470218.2014.982664

Leggett NC, Thomas NA, Loetscher T, and Nicholls MER. 2013. The Life of P: "Just Significant" Results Are on the Rise. *Quarterly Journal of Experimental Psychology* 66:2303-2309. 10.1080/17470218.2013.863371

Light RJ, and Pillemer DB. 1984. *Summing Up: The Science of Reviewing Research*. Cambridge, Mass.: Harvard University Press.

Macaskill P, Walter SD, and Irwig L. 2001. A Comparison of Methods to Detect Publication Bias in Meta-Analysis. *Statistics in Medicine* 20:641-654. 10.1002/sim.698

Masicampo EJ, and Lalande DR. 2012. A Peculiar Prevalence of P Values just Below .05. *Quarterly Journal of Experimental Psychology* 65:2271-2279. 10.1080/17470218.2012.711335

McShane BB, Bockenholt U, and Hansen KT. 2016. Adjusting for Publication Bias in Meta-Analysis: An Evaluation of Selection Methods and Some Cautionary Notes. *Perspectives on Psychological Science* 11:730-749. 10.1177/1745691616662243

Paldam M. 2015. Simulating an Empirical Paper by the Rational Economist. *Empirical Economics*:1-25. 10.1007/s00181-015-0971-6

Reed WR. 2015. A Monte Carlo Analysis of Alternative Meta-Analysis Estimators in the Presence of Publication Bias. *Economics-the Open Access Open-Assessment E-Journal* 9. 10.5018/economics-ejournal.ja.2015-30

Renkewitz F, and Keiner M. 2016. How to Detect Publication Biases from Published Data? A Monte Carlo Simulation of Different Methods. 50 Kongress der Deutschen Gesellschaft für Psychologie. Leipzig.

Simmons JP, and Simonsohn U. 2017. Power Posing. *Psychological Science*:0956797616658563. 10.1177/0956797616658563

Simonsohn U, Nelson LD, and Simmons JP. 2014a. P-Curve and Effect Size: Correcting for Publication Bias Using Only Significant Results. *Perspectives on Psychological Science* 9:666-681. 10.1177/1745691614553988

Simonsohn U, Nelson LD, and Simmons JP. 2014b. P-curve: A Key to the File Drawer. *Journal of Experimental Psychology-General* 143:534-547. 10.1037/a0033242

Simonsohn U, Simmons JP, and Nelson LD. 2015. Better P-Curves: Making P-Curve Analysis More Robust To Errors, Fraud, and Ambitious P-Hacking, A Reply To Ulrich and Miller (2015). *Journal of Experimental Psychology-General* 144:1146-1152. 10.1037/xge0000104

Stanley TD. 2017. Limitations of PET-PEESE and Other Meta-Analysis Methods. *Social Psychological and Personality Science*:1948550617693062. 10.1177/1948550617693062

Stanley TD, and Doucouliagos H. 2014. Meta-Regression Approximations to Reduce Publication Selection Bias. *Research Synthesis Methods* 5:60-78. 10.1002/jrsm.1095

Tang JL, and Liu JLY. 2000. Misleading Funnel Plot for Detection of Bias in Meta-Analysis. *Journal of Clinical Epidemiology* 53:477-484. 10.1016/s0895-4356(99)00204-8

van Aert RCM, Wicherts JM, and van Assen MALM. 2016. Conducting Meta-Analyses Based on p Values. *Perspectives on Psychological Science* 11:713-729. 10.1177/1745691616650874

van Assen MALM, van Aert RCM, and Wicherts JM. 2015. Meta-Analysis Using Effect Size Distributions of only Statistically Significant Studies. *Psychological Methods* 20:293-309. 10.1037/met0000025 10.1037/met0000025.supp (Supplemental)
